# Exploring predictors of COVID-19 precautionary behaviors during the initial rollout of COVID-19 vaccines in a predominately Hispanic sample

**DOI:** 10.1016/j.bbih.2024.100870

**Published:** 2024-09-19

**Authors:** Gabriel A. Frietze, Margie E. Padilla, Amanda M. Loya, Alyssa A. Martinez, Amir G. Hernandez, José O. Rivera

**Affiliations:** a The University of Texas at El Paso, School of Pharmacy, 500 W. University, El Paso, TX, 79968, USA; b The University of Texas at El Paso, Interdisciplinary Health Sciences, 500 W. University, El Paso, TX, 79968, USA

## Abstract

• Precautionary behaviors were lower if primary language in household was Spanish.
• Testing positive for COVID-19 was inversely associated with precautionary behaviors.
• Healthcare practitioners were the most trusted source of COVID-19 information.

## Informed consent statement

Informed consent was obtained from all subjects involved in the study.

## 1 Introduction

After first emerging in an outbreak in Wuhan, China in 2019, the severe acute respiratory syndrome coronavirus 2 (SARS-CoV-2) quickly became a public health crisis of epic proportions. The coronavirus disease 2019 (COVID-19), as it was later called, was officially declared a global pandemic by the World Health Organization on March 11, 2020 (Cucinotta and Vanelli, 2020). The first case reported in El Paso, Texas occurred on March 13, 2020, in a Hispanic male in his 40's (Montes and Dearman, 2020). By May 4, 2020, the virus had infected over 1000 individuals (9.2%); 13,000 cases were surpassed by July 31, 2020 (City of El Paso, 2020a) and 100,000 cases were reached by January 31, 2021 (City of El Paso, 2021). In early November of 2020, active cases surpassed 29,743 with rates of hospitalized patients reaching 1148 patients from Nov. 6–12, 2020 (City of El Paso, 2020b). Like other communities of color across the U.S., El Paso experienced a disproportionate impact of the pandemic.

Progress made towards eliminating inequities in social determinants of health (SDoH) in Hispanic/Latinx populations have regressed since the emergence of the novel SARS-CoV2 (COVID-19) pandemic in 2019. According to the Centers for Disease Control and Prevention (CDC) (updated July 2022); Hispanics/Latinx are 1.5x more likely to contract COVID-19; 1.8x more likely to be hospitalized; and 1.7x more likely to die from COVID-19 related complications compared to white/non-Hispanic populations (CDC, 2023). The increased risk of death due to COVID-19 is further supported by a recent study that outlined Hispanic/Latinx substantial decline in life expectancy (−1.47) compared to Black (−0.62) and White/non-Hispanic populations (−0.44) (Andrasfay and Goldman, 2021).

The increase in COVID-19 related deaths, hospitalizations, and risk for acquiring COVID-19 do not accurately capture the indirect impact of COVID-19 on Social Determinants of Health (SDoH) in the Hispanic/Latinx population. Recent trends demonstrate and have highlighted reasons that further increase the gap in SDoH. Hispanic/Latinx populations have disproportioned burden of chronic comorbidities (e.g., diabetes, asthma, COPD, obesity) that place them at higher risks for COVID infection, hospitalization, and death. Additionally, Hispanics/Latinx populations located in urban centers live in crowded conditions and/or multigeneration households that remove the ability for appropriate social distancing. Additionally, social distancing is difficult when minorities tend to be employed in first line occupations that place them directly interacting face to face with the general population (Hooper et al., 2020). The complexity of COVID-19 on the growing health disparity gap warrants further exploration and understanding.

Martin et al. (2023) conducted a mixed methods study in 255 Hispanic/Latinx individuals which assessed various barriers, preventive measures, and practices during the COVID-19 pandemic. The majority (72.2% female) of the sample reported good preventive practices: masking (98.4%), social distancing (96.1%), and not going to work if COVID positive (95.3%). However, participants also reported an increase in job, food, and housing insecurities as a result of the pandemic. Specifically, when comparing individuals with job insecurity and individuals without job insecurity during the pandemic, participants with job insecurity were more likely to experience food insecurity (73% vs. 39%, *p* < 0.0001), housing problems (44% vs. 14%, *p* < 0.0001), and eviction threats (55% vs. 9%, *p* < 0.0001). These findings highlight the complex interplay between preventive practices and socioeconomic vulnerabilities within Hispanic/Latinx individuals during the pandemic. The Hispanic/Latinx population is the largest minority group, comprising of 18.7% of the total US population (Bureau, 2022). In Texas the Hispanic/Latinx population makes up 39.3% of the total state population (Bureau, 2022). Locally in the El Paso Metroplex, approximately 83% of the city population identifies as Hispanic/Latinx (US Census Bureau Quick Facts, n.d.).

In response to the critical need to improve COVID-19 outcomes in Hispanics, the University of Texas at El Paso (UTEP) COVID-19 Taskforce was created to establish a COVID-19 vaccination clinic at UTEP during the initial roll out of the COVID-19 vaccines. A Chief Medical Officer and an operations manager were appointed for the COVID-19 vaccination clinic and an application was submitted and received approval from the Texas Department of Human and Health Services. The logistics of the clinic were separate for clinical, legal, operations, technology, and communications domains. The UTEP School of Pharmacy (SoP) was responsible for receiving, storing, inventory control, transportation, preparation, dispensing, administration, education, and monitoring of possible adverse drug reactions. Additionally, the SoP faculty, staff, and students were scheduled to provide the distribution and clinical activities of the clinic. Here, faculty and students from the SoP, the UTEP College of Health Sciences Clinical Laboratory Sciences, the UTEP Public Health program, and the UTEP School of Nursing, participated in several clinical activities. To ensure that a systematic procedure was in place, a handbook of operation procedures was developed and implemented which included training modules for staff, faculty, and student volunteers. Lastly, the clinic was divided into five stations: 1) receiving and storing of vaccines, 2) prescreening, informed consent, and registration of participants, 3) preparation of vaccine, 4) screening and administration of vaccine, and 5) observation and education post vaccination. Implementing the UTEP COVID-19 Vaccination Clinic to increase vaccination in our predominately Hispanic, underserved community of El Paso, served as a critical step towards reducing barriers accessing COVID-19 vaccines.

In addition to improving access to COVID-19 vaccines, we sought to concurrently understand community perceptions about COVID-19. A community survey was developed to examine COVID-19 attitudes and perceptions within individuals who were vaccinated at the UTEP COVID-19 Vaccination Clinic. The purpose of the community survey was to investigate perceptions towards COVID-19 during the initial rollout of the COVID-19 vaccines in recently vaccinated community members. We assessed a variety of factors including trusted sources of COVID-19 information (e.g., healthcare practitioners), COVID-19 perceptions (e.g., perceived severity), engagement in COVID-19 precautionary behaviors (e.g., wearing a face mask), and intentions to engage in precautionary behaviors after being vaccinated.

## 2 Materials and methods

### 2.1 Participants

A total of 4494 adults (*M*_age_ = 32.69, *SD* = 25.56; 56.3% female) who were recently vaccinated with a COVID-19 vaccine at the UTEP COVID-19 vaccine clinic completed a brief 10–15 min survey. To be included in the study, participants needed to speak English and be over the age 18. The average time to complete the survey was approximately 653.25 s (approximately 11 min) with a standard deviation equal to 693.21. The time to complete the survey was indexed by the amount of time spent with the survey link open (which includes the 2 min consent form). Initially, a total of 6521 community members opened the survey link and either read the consent form or initiated the survey. A large proportion of participants dropped out of the survey quickly after beginning. For example, 732 participants did not make it past the 7th question of the survey. Exclusion criteria was determined to enhance the quality of our data. For example, we included a cutoff criterion and excluded data from participants who had the survey link open for less than 6 min. The 6 min cut-off was identified as a reasonable cutoff criterion given that it is unlikely for participants to have read and agreed to the consent form as well as complete the survey in under 6 min. The 6 min cut-off was intended to improve the quality of our data by screening out participants who did not complete the survey or who may have deliberately rushed through the survey. Additionally, we excluded participants for not answering or failing to respond correctly to an attention check item that was embedded within the online survey (*n* = 4). Moreover, we excluded participants who did not report receiving the COVID-19 vaccine (*n* = 139). The latter participants were excluded as it was unexpected to have unvaccinated participants in the study considering that everyone was recruited from the waiting area of the COVID-19 immunization clinic, where vaccinated persons were being monitored by medical professionals in case there was an adverse event. We believe that participants might have misinterpreted the COVID-19 vaccine status question as having previously received the COVID-19 vaccine “prior to today”. A total of 2027 participants were excluded utilizing the 6-min cut-off criterion, the attention check, and COVID-19 vaccine status. The remaining 4494 adults were included in the analysis. Recruitment and participation of subjects was completed in accordance with IRB guidelines for human subjects and all data was collected anonymously with no identifiers.

### 2.2 Measures

A variety of measures were included in the current study including a demographics and background survey, items to examine perceptions about COVID-19 (e.g., perceived susceptibility), vaccine myths (e.g., vaccines can cause autism), and a series of other related measures. See Table 1 for a complete list of measures. The full survey (excluding the consent form) is also available as a supplementary material.

**Table 1:** Measures included in the survey.

Variable Name	Description	Sample Item(s)
Basic Demographics and Background Survey	9-items assessing basic demographics such as age, gender, ethnicity, primary language spoken at home and household size.	*“What is your age in years?”* *“What is your primary language spoken at home?”* Response options included (1) English, (2) Spanish.

Social Media Sources of Health Information	A single item assessed social media sources of health information adapted from Frietze et al. (2023) and Allington et al. (2020).	“*If you obtain health information online or through social media, what sources do you use? (Select all that apply)*”. Thirteen response options were provided: “*Facebook”, “Google”, “Groupme”, “Instagram”, “LinkedIn”, “Reddit”, “Snapchat”, “TikTok”, “Twitter”, “WebMd”, “Whatsapp”, “Yahoo”,* and *“other”*.
COVID-19 Vaccine Uptake	A single item assessing COVID-19 vaccination status.	**“***Have you received the COVID-19 vaccine?”* Response options included (1) yes, (2) no. Note: Given that all participants were recruited in a waiting area after vaccination, those who did not answer yes were eliminated from the analyses

Underlying Health Conditions	A single item assessed underlying health conditions.	*“Do you have any of the following underlying health conditions? If so, please select all that apply.”* Ten response options were provided: “*autoimmune”, “asthma”, “COPD”, “diabetes”, “high blood pressure”, “heart condition”, “smoker”, “prefer not to answer”, “I do not have any underlying health conditions”,* and another option which permitted text entry.
Ever Tested Positive for COVID-19	A single item assessed having ever tested positive for COVID-19.	*“Have you tested positive for COVID-19?”* Response options included (1) yes, (0) no.
COVID-19 Symptoms	A single item assessed the symptoms experienced in those who reported having tested positive for COVID-19.	*“What were your symptoms?”* Eighteen response options were provided: *“anxiety”, “body aches”, “depression”, “diarrhea”, “difficulty breathing”, “dry cough”, “fatigue”, “fever”, “headache”, “loss of smell”, “loss of taste”, “nasal congestion”, “nausea”, “pink eye”, “rash”, “sore throat”, “vomiting”,* and another option which permitted text entry.
Severity of COVID-19 Symptoms	A single item assessed the severity of COVID-19 symptoms among participants who reported testing positive for COVID-19.	*“How severe were your symptoms for COVID-19?”* Response options included: 1) no symptoms, 2) slightly severe, 3) somewhat severe, and 4) extremely severe. This item was excluded from regression analyses given that it was only asked to participants who reported having tested for positive for COVID-19.
Hospitalized for COVID-19 Complications	A single item assessed ever being admitted to the hospital for COVID-19 complications.	*“Where you admitted to the hospital for COVID-19 complications?”* Response options included (1) yes, (2) no. This variable was excluded from the regression analyses as only 2.6% of the sample responded yes (see Table 2).
Family or Friend Hospitalized for COVID-19 Complications	A single item assessed having a family or friend who has been admitted to the hospital for COVID-19 complications.	*“Do you have close family members or friends who have been admitted to the hospital for COVID-19 complications?”* Response options included (1) yes, (2) no.
Family or Friend Deceased Due to COVID-19	A single item assessed having a family or friend who has died due to COVID-19.	*Do you have close family members or friends who have passed away due to COVID-19?”* Response options included (1) yes, (2) no.
Intentions to Vaccinate Child/Children	A single item assessed parent's intentions to vaccinate their child/children.	“*If you have a child, do you intend to get your child/children vaccinated against COVID-19?”* Response options included: 0 = No, 1 = Yes, 2 = not applicable, 3 = prefer not to answer, and 4 = other. The “not applicable”, “prefer not to answer”, and “other” response options were excluded in data analysis. This factor was not included within regression analyses as it was only asked to parents.
Essential Worker	A single item assessed if participants are essential workers.	**“***Are you considered an essential worker/personnel?”* Response options included (1) yes, (2) no.

Current Medication Status	A single item assessed medication status.	*“Do you and/or your family currently take medications for any health conditions?”* Response options included (1) yes, (2) no.
Medication Access	A single item assessed medication access during the pandemic.	*“Have you and/or your family been able to purchase your medications?* Response options included (1) yes, (2) no.
Meal Assistance	A single item assessed if participants received meal assistance during the pandemic.	“*Have you received meal assistance from your local______?”* Seven response options were provided: “*Faith-based organization”, “family”, “friends”, “food bank”, “non-faith-based organizations”, “UTEP food pantry”,* and an *“not applicable”*.

Facemask Frequency	A single item assessed frequency of wearing a facemask.	*“How often do you wear your facemask when in public places?”* Response options included (1) Never, (2) Once in a while, (3) About half the time, (4) Most of the time, and (5) Always.
COVID-19 Testing Frequency	A single item assessed frequency of testing for COVID-19.	*“How often do you test for COVID-19?* Response options included (1) never, (2) monthly, (3) once a week, (4) twice a week, and (5) three times a week.
Personal Health Importance	Three-items from Clark et al. (2020) assessed personal health importance.	“*I am concerned about my health and am taking action to prevent COVID-19”, “My health is my top priority”,* and *“Taking care of my health means a lot to me.”* Response options ranged from (1) strongly disagree, to (5) strongly agree. A composite was created by averaging the three items.
Perceived Susceptibility of COVID-19	Five-items from Clark et al. (2020) assessed perceived susceptibility.	“*I am less likely than most people to get COVID-19”, “I am not at risk for getting infected with COVID-19”, “My body could fight off COVID-19 infection”, “People like me don't get COVID-19”,* and *“There is little chance that I could get or spread COVID-19 from what I do in my everyday life.”* Response options ranged from (1) strongly disagree, to (5) strongly agree. A composite was created by averaging the five items.
Perceived Efficacy of Behavioral Health Precautions	Five-items from Clark et al. (2020) assessed assumptions regarding the efficacy of behavioral health precautions.	“*Avoiding crowds is an effective method for fighting COVID-19”, “Practicing social distancing is an effective method for avoiding COVID-19”, “Staying at home is an effective method for avoiding COVID-19”, “Washing your hands frequently is an effective method for avoiding COVID-19”,* and *“Wearing a surgical mask is an effective method for avoiding COVID-19.”* Response options ranged from (1) strongly disagree, to (5) strongly agree. A composite was created by averaging the five items.
Trusted COVID-19 Information Sources	A single item assessed the number of COVID-19 informational sources one has encountered.	“*If you were receiving information about COVID-19 and the COVID-19 vaccine, who would you believe is the most credible? (Select all that apply)”. Response Options:* “*Healthcare practitioner (e.g., pediatrician, family practice doctor)*”, “*communication health clinic*”, “*Pharmacy (e.g., Walgreens, CVS, etc.,)*”, “*Pharmacist*”, “*school nurse*”, “*family/friends*”, “*television*”, “*social media*”, “*Government website (e.g. CDC, FDA, etc.)*”, “*World Health Organization (WHO)*”, “*television*”, “*radio*”, “*internet*”, “*newspaper*”, *“Community health worker or promotor(a) de salud”, “elected officials”, “public health official”, “university communications”,* and an *“other”* option which permitted text entry.
Attention Check	A single item served as an attention check.	“*If you are paying attention, please select “Blue” for the following response.”* Response options included: (1) Red, (2) Green, (3) Blue, and (4) Yellow

Perceived Effectiveness of the COVID-19 Vaccine	Two items assessed perceived effectiveness of the COVID-19 vaccine at preventing infection and sickness.	*“I believe the COVID-19 vaccine will be effective in preventing the infection”* and *“I believe if I get the COVID-19 vaccine, I will be less likely to get sick.”* Response options ranged from (1) strongly disagree, to (5) strongly agree. A composite was created by averaging the two items.
Fear of COVID-19 Vaccine Side-Effects	Three items assessed fear of COVID-19 vaccine side-effects.	*“I worry that the COVID-19 vaccine might negatively affect me”, “I worry about the short-term side effects of the COVID-19 vaccine”,* and *“I worry that the COVID-19 vaccine might have unknown long-term side effects.”* Response options ranged from (1) strongly disagree, to (5) strongly agree. A composite was created by averaging the three items.

Perceived Harm of the COVID-19 Vaccine	Two items assessed perceived harms of the COVID-19 vaccine.	*“I think the COVID-19 vaccine may cause health problems in the future”* and *“I think the COVID-19 vaccine is unsafe.”* Response options ranged from (1) strongly disagree, to (5) strongly agree. A composite was created by averaging the two items.
Belief that COVID-19 Vaccines Cause Mild-Side Effects	A single item assessed perceived mild side-effects of the COVID-19 vaccine.	*“I think the COVID-19 vaccine might cause short-term problems like fever or discomfort.”* Response options ranged from (1) strongly disagree, to (5) strongly agree.

Perceived Safety of Vaccines	A single item from Frietze et al. (2023) assessed perceived safety of vaccines.	*“I believe vaccines are safe and effective.”* Response options ranged from (1) strongly disagree, to (5) strongly agree.
Perceived Safety of Moderna COVID-19 Vaccine	A single item adapted from Frietze et al. (2023) assessed perceived safety of the Moderna COVID-19 vaccine.	*“I believe the Moderna COVID-19 vaccine is safe and effective.”* Response options ranged from (1) strongly disagree, to (5) strongly agree.
Perceived Safety of Pfizer COVID-19 Vaccine	A single item adapted from Frietze et al. (2023) assessed perceived safety of the Pfizer COVID-19 vaccine.	*“I believe the Pfizer COVID-19 vaccine is safe and effective.”* Response options ranged from (1) strongly disagree, to (5) strongly agree.
General Vaccine Support	A single item assessed vaccine support.	*“I believe everyone should be immunized against serious diseases.”* Response options ranged from (1) strongly disagree, to (5) strongly agree.
COVID-19 Vaccine Support	A single item assessed positive COVID-19 vaccine beliefs.	*“I believe everyone should be immunized against COVID-19.” Response options ranged from (1) strongly disagree, to (5) strongly agree.*
Vaccine Autism Myth	A single item assessed if participants believed vaccines cause autism.	*“I believe vaccines cause autism.”* Response options ranged from (1) strongly disagree, to (5) strongly agree.

Risk Perceptions of COVID-19 Infection	Two items from Taghrir et al. (2020) assessed risk perceptions of COVID-19.	*“I may become infected with COVID-19 more easily than others”* and *“I am afraid to be infected with COVID-19.”* Response options ranged from (1) strongly disagree, to (5) strongly agree. A composite was created by averaging the two items.
Perceived Severity of SARS-CoV-2 Infection	A single item assessed perceived severity of COVID-19 infection.	*“How severe do you think the COVID-19 infection is for yourself?” Response options ranged from (1) strongly disagree, to (5) strongly agree.*

Past Health Precautionary Behaviors	Four-items from Clark et al. (2020) assessed health precaution behaviors over the past 3 months (i.e., preceding vaccination).	“*Over the past* 3 months*, I have practiced social distancing to avoid COVID-19”, “Over the past* 3 months*, I have stayed at home to avoid COVID-19”, “Over the past* 3 months*, I have washed my hands frequently to avoid COVID-19”,* and *“Over the past* 3 months*, I wore a mask to avoid COVID-19.”* Response options ranged from (1) strongly disagree, to (5) strongly agree. A composite was created by averaging the four items.
Future Intentions to Engage in Health Precautionary Behaviors	Four-items adapted from Clark et al. (2020) assessed health precaution behaviors in the upcoming 3 months (i.e., preceding vaccination).	“*In the upcoming* 3 months*, I have practiced social distancing to avoid COVID-19”, “In the upcoming* 3 months*, I have stayed at home to avoid COVID-19”, “In the upcoming* 3 months*, I have washed my hands frequently to avoid COVID-19”,* and *“In the upcoming* 3 months*, I wore a mask to avoid COVID-19.”* Response options ranged from (1) strongly disagree, to (5) strongly agree. A composite was created by averaging the four items.

### 2.3 Procedure

Flyers were posted within the observation room of the university COVID-19 vaccine clinic recruiting participants to complete a short 10–15-min survey on their own electronic device (e.g., cell phone, laptop, tablet). Importantly, participants were being monitored in an observation room and asked to remain in the room for a minimum of 15 min to ensure that they did not have adverse effects after receiving the COVID-19 vaccine. During this time, participants completed the survey. The online survey platform QuestionPro© was used to collect data. Protocol administration was conducted January–May 2021.

### 2.4 Approach to analysis

Descriptive statistics were calculated for demographic variables such as age, ethnicity, gender, and educational level. Two multiple linear regression models examined predictors of past health precautionary behaviors (i.e., 3 months preceding vaccination) and future intentions to engage in health precautionary behaviors (i.e., 3 months following vaccination). Preliminary analyses were conducted to ensure no serious violations of the assumptions of normality, linearity, multicollinearity, and homoscedasticity. The assumption of multicollinearity was not violated according to the collinearity statistics (i.e., Tolerance and VIF). All variables entered in the model had a VIF well below 4 and only four of the independent variables entered in each regression model had a VIF that were above 3 (e.g., ranging from 3.03 to 3.42). In the first regression model, the dependent variable was past health precautionary behaviors and twenty-four independent variables included in the model: 1) age, 2) primary language, 3) gender identity, 4) Ever Tested Positive for COVID-19, 5) Family or Friend Hospitalized for COVID-19 Complications, 6) Family or Friend Deceased Due to COVID-19, 7) Essential Worker, 8) Facemask Frequency, 9) COVID-19 Testing Frequency, 10) Personal Health Importance, 11) Perceived Susceptibility of COVID-19, 12) Perceived Efficacy of Behavioral Health Precautions, 13) Perceived Effectiveness of the COVID-19 Vaccine, 14) Fear of COVID-19 Vaccine Side-Effects, 15) Perceived Harms of the COVID-19 Vaccine, 16) Perceived COVID-19 Vaccine Mild-Side Effects, 17) Perceived Safety of Vaccines, 18) Perceived Safety of Moderna COVID-19 Vaccine, 19) Perceived Safety of Pfizer COVID-19 Vaccine, 20) Vaccine Support, 21) COVID-19 Vaccine Support, 22) Vaccine Autism Myth, 23) Risk Perceptions of COVID-19 Infection, and 24) Perceived Severity of SARS-CoV-2 Infection. The second regression model included the same 24 independent variables and replaced the dependent variable with future intentions to engage in health precautionary behaviors.

## 3 Results

### 3.1 Demographics and background

The COVID-19 vaccination clinic at UTEP administered a total of 30,130 COVID-19 doses between January and May 2021. The sample was predominately Hispanic (*n* = 3662; 81.5%) and nearly half (*n* = 1867; 41.5%) reported that Spanish was their primary language spoken at home. More than a third of the sample (35%) reported that their family annual income was below $40,000. Most of the sample (91.4%) reported that their household size was two or more and 46.2% of the sample reported that their household size was four or more. See Table 1 for demographic and background information.

### 3.2 COVID-19 symptoms and history

Approximately 18% of the participants reported having previously tested positive for COVID-19 (see Table 2). Among those who reported to have tested positive for COVID-19, the five most common reported symptoms reported were: headache (*n* = 506; 62.7%), fatigue (*n* = 491; 60.8%), loss of smell (*n* = 477; 59.1%), loss of taste (*n* = 446; 55.3%), and body aches (*n* = 442; 54.8%); see Table 2 for the complete list of symptoms. Importantly, another option permitted text entry for “other symptoms” and a number of participants reported swollen body parts (e.g., lips, face, limbs), one participant reported that they suffered a heart attack due to COVID-19, and one participant reported “none so far”, which may suggest that they were COVID-19 positive during the time they were completing the survey in the observation room of the clinic and they may be anticipating symptoms to emerge. Most of the participants reported that their COVID-19 symptoms were slightly severe (*n* = 475; 56.6%) and 129 (16%) reported to have no symptoms (see Table 2). Twenty-one participants reported to have been hospitalized for COVID-19 complications at some point and 1157 participants reported to have family or friends who were hospitalized for COVID-19 complications. Nearly a quarter of the sample (*n* = 1102; 24.5%) reported to have a family or friend who was deceased due to COVID-19. During the time of the study, the COVID-19 vaccine had not been released for children, however, participants were asked, “*If you have a child, do you intend to get your child/children vaccinated against COVID-19*?” A quarter of the sample (25.5%; *n* = 1145) reported that they intend to vaccinate their child with the COVID-19 vaccine, 4.2% (*n* = 187) reported “no”, 68.1% (*n* = 3062) reported “not applicable”, 2.0% (*n* = 88) reported “prefer not to answer”, and the remaining responses were missing (0.3%; *n* = 12). After removing the participants who reported “not applicable”, suggesting that they do not have children the proportion of parents who intend to vaccinate their children is equal to 79.96%.

**Table 2:** Demographic and background information (N = 4494).

Variable	Total Responses	Percentage of Responses
Gender Identity		
Male	1894	42.1%
Female	2528	56.3%
Transgender Female	6	0.1%
Transgender Male	4	0.1%
Gender Variant/Non-Conforming	39	0.9%
Prefer not to answer	13	0.3%
Other	3	0.1%
Missing	7	0.2%

Ethnicity		
Non-Hispanic	748	16.6%
Hispanic	3662	81.5%
Prefer not to answer	51	1.1%
Missing	33	0.7%

aRace		
White	3412	75.9%
Black	120	2.7%
Asian	162	3.6%
Pacific Islander	17	0.4%
Native-American	120	2.7%
Other	616	13.7%
Prefer not to answer	227	5.1%

Primary Language		
English	2510	55.9%
Spanish	1867	41.5%
Other	105	2.3%
Missing	12	0.3%

Household income		
Less than $5000	199	4.4%
$5001-$20,000	530	11.8%
$20,001-$40,000	844	18.8%
$40,001-$60,000	745	16.6%
$60,001-$80,000	525	11.7%
$80,001-$100,000	380	8.5%
$100,001 or more	608	13.5%
Do not know	403	9.0%
Prefer not to answer	259	5.8%
Missing	1	0.1%

Average Household Size		
One	379	8.4%
Two	957	21.3%
Three	1073	23.9%
Four	1155	25.7%
Five	633	14.1%
Six	216	4.8%
Seven or more	74	1.6%
Missing	7	0.2%

aSocial Media Sources of Health Information		
Facebook	1811	40.3%
Google	3061	68.1%
Groupme	136	3.0%
Instagram	1610	35.8%
LinkedIn	284	6.3%
Reddit	363	8.1%
Snapchat	522	11.6%
Tik-Tok	625	13.9%
Twitter	1029	22.9%
WebMd	1082	24.1%
Whatsapp	518	11.5%
Yahoo	343	7.6%
Other	449	10.0%

aTrusted COVID-19 Informational Sources		
Healthcare practitioner	3912	87.0%
Community health Clinic	2270	50.5%
Pharmacist	1860	41.4%
Pharmacy (e.g., Walgreens, CVS, etc.)	1247	27.7%
School nurse	856	19.0%
Family/friends	267	5.9%
Social media	156	3.5%
Social worker	180	4.0%
Government website (e.g., CDC, FDA etc.)	2796	62.2%
World Health Organization (WHO)	2810	62.5%
Radio	156	3.5%
Television	313	7.0%
Internet	338	7.5%
Newspaper	293	6.5%
Community health worker or promotor(a) de salud	977	21.7%
Elected officials	152	3.4%
Public health officials	2527	56.2%
University communications	1387	30.9%
Other	81	1.8%

aUnderlying Health Conditions		
Autoimmune	119	2.6%
Asthma	530	11.8%
COPD	18	0.4%
Diabetes	254	5.7%
High Blood Pressure	558	12.4%
Heart Condition	110	2.4%
Smoker	300	6.7%
Prefer not to answer	173	3.8%
No underlying health conditions	2507	55.8%
Other	329	7.3%

Received COVID-19 Vaccine		
Yes	4494	97.0%
No (Excluded from analysis)	119	2.6%
Prefer not to answer (Excluded from analysis)	6	0.1%
Missing (Excluded from analysis)	14	0.3%

Ever Tested Positive for COVID-19		
Yes	807	18.0%
No	3667	81.6%
Missing	20	0.4%

aCOVID-19 Positive Symptoms (*n* = 807)		
Anxiety	160	19.8%
Body Aches	442	54.8%
Depression	102	12.6%
Diarrhea	208	25.8%
Difficulty breathing	212	26.3%
Dry cough	296	36.7%
Fatigue	491	60.8%
Fever	319	39.5%
Headache	506	62.7%
Loss of smell	477	59.1%
Loss of taste	446	55.3%
Nasal congestion	365	45.1%
Nausea	148	18.3%
Pink eye	14	1.7%
Rash	30	3.7%
Sore throat	298	36.9%
Vomiting	52	6.4%
Other	62	7.7%

Severity of COVID-19 Symptoms (*n* = 822)		
No symptoms	143	17.40%
Slightly severe	459	55.84%
Somewhat severe	189	22.99%
Extremely severe	31	3.77%

Hospitalized for COVID-19 Complications (*n* = 807)		
Yes	21	2.6%
No	780	96.7%
Missing	6	0.7%

Family or Friends Hospitalized for COVID-19 Complications		
Yes	1157	25.7%
No	3327	74.0%
Missing	10	0.2%

Family or Friends Deceased Due to COVID-19		
Yes	1102	24.5%
No	3375	75.1%
Missing	17	0.4%

If you have a child, to you intend to vaccinate them against COVID-19		
Yes	1145	25.5%
No	187	4.2%
Not applicable	3062	68.1%
Prefer not to answer	88	2.0%
Missing	12	0.3%

Do you and/or your family currently take medications for any health conditions?		
Yes	2441	54.3%
No	2027	45.1%
Missing	26	0.6%

Have you and/or your family been able to purchase your medications?		
Yes	2241	49.9%
No	97	2.2%
Not applicable	123	2.7%
Missing	2033	45.2%

aHave you received meal assistance from your local ________.		
Faith-based organization	110	2.4%
Family	214	4.8%
Friends	120	2.7%
Food bank	290	6.5%
Non faith-based organization	32	0.7%
UTEP food pantry	33	0.7%
Not applicable	1962	43.7%

Essential Worker/Personnel		
Yes	1757	39.1%
No	2695	60.0%
Missing	42	0.9%

Note.

a Indicates a variable which includes response options that do not total to 100% as these variables included a “select all that apply” response.

### 3.3 Trusted sources of COVID-19 information

The most trustworthy sources of COVID-19 information were healthcare practitioners (*n* = 3912; 87%), the World Health Organization (WHO) (2810; 62.5%), and government websites (e.g., CDC, FDA, etc.) (*n* = 2810; 62.2%). Elected officials (*n* = 152; 3.4%), social media (*n* = 156; 3.5%) and radio (*n* = 156; 3.5%) were reported as the least trustworthy sources of COVID-19 information. Despite social media predominately being reported as not trusted for COVID-19 information, for general health information the top social media sources participants used included: Google (*n* = 3061; 68.1%), Facebook (*n* = 1811; 40.3%), and Instagram (*n* = 1610; 35.8%).

### 3.4 Predictors of past health precautionary behaviors

A multiple linear regression analysis was performed to predict past health precautionary behaviors from 24 variables (see Table 3). The overall model predicted past health precautionary behaviors, (*F* (24, 3856) = 80.40, *p* < 0.001), with an *R*^2^ of 0.34. Thirteen of the 24 variables emerged as predictors of past health precautionary behaviors. The following variables were positively associated with future intentions to engage in health precautionary behaviors: language (English = 1, Spanish = 0), essential worker (coded as Yes = 1, No = 2), facemask frequency, personal health importance, perceived susceptibility of COVID-19, perceived efficacy of behavioral health precautions, perceived safety of vaccines, general vaccine support, COVID-19 vaccine support, risk perceptions of COVID-19, and perceived severity of COVID-19. Variables that were inversely associated with future intentions to engage in health precautionary behaviors include having ever tested positive for COVID-19 (coded as Yes = 1, No = 0), and vaccine autism myth (see Table 3).

**Table 3:** Summary of multiple linear regression analysis for variables predicting past health precautionary behaviors (n = 3893).

Variables	*B*	*SE B*	β	*t*	95% confidence intervals		*P*
					Lower	Upper	
Constant	.254	.166		1.536	−.070	.579	.125
Age	.001	.000	.026	1.917	.000	.001	.055
Primary Language (1 = English, 0 = Spanish)	.106	.016	.091	6.678	.075	.138	<.001
Gender (1 = Male, 0 = Female)	−.029	.016	−.025	−1.786	−.060	.003	.074
Ever Tested Positive for COVID-19 (1 = Yes, 0 = No)	−.163	.020	−.109	−7.986	−.203	−.123	<.001
Family or Friend Hospitalized for COVID-19 Complications	.024	.020	.018	1.167	−.016	.063	.243
Family or Friend Deceased Due to COVID-19	−.037	.021	−.027	−1.786	−.077	.004	.074
Essential Worker	.068	.016	.057	4.270	.037	.099	<.001
Facemask Frequency	.298	.026	.154	11.300	.246	.349	<.001
COVID-19 Testing Frequency	.003	.010	.004	.265	−.016	.021	.791
Personal Health Importance	.085	.011	.113	7.968	.064	.106	<.001
Perceived Susceptibility of COVID-19	.037	.012	.046	3.102	.014	.060	.002
Perceived Efficacy of Behavioral Health Precautions	.268	.015	.270	17.751	.239	.298	<.001
Perceived Effectiveness of the COVID-19 Vaccine	.002	.012	.003	.196	−.022	.027	.845
Fear of COVID-19 Vaccine Side-Effects	.014	.012	.023	1.241	−.008	.037	.215
Perceived Harm of the COVID-19 Vaccine	−.018	.015	−.024	−1.167	−.047	.012	.243
Belief that COVID-19 Vaccines Cause Mild-Side Effects	−.002	.009	−.003	−.231	−.020	.016	.818
Perceived Safety of Vaccines	.032	.014	.040	2.356	.005	.059	.019
Perceived Safety of Moderna COVID-19 Vaccine	.028	.017	.039	1.682	−.005	.061	.093
Perceived Safety of Pfizer COVID-19 Vaccine	.009	.019	.011	.455	−.028	.045	.649
General Vaccine Support	.048	.020	.058	2.405	.009	.088	.016
COVID-19 Vaccine Support	.043	.019	.054	2.228	.005	.081	.026
Vaccine Autism Myth	−.047	.011	−.067	−4.334	−.068	−.026	<.001
Risk Perceptions of COVID-19 Infection	.049	.011	.070	4.467	.028	.071	<.001
Perceived Severity of SARS-CoV-2 Infection	.045	.008	.086	5.611	.030	.061	<.001

*Note*. Sample size is less than 4494 due to missing data. Gender was dichotomized in this analysis as 1 = Male, 0 = Female. Primary language was dichotomized as 1 = English, 0 = Spanish. Ever tested positive for COVID-19 was coded as 1 = Yes, 0 = No.

### 3.5 Predictors of future intentions to engage in health precautionary behaviors

A multiple linear regression analysis was performed to predict future intentions to engage in health precautionary behaviors from 24 variables (see Table 4). The overall model predicted future intentions to engage in health precautionary behaviors, (*F* (24, 3753) = 75.04, *p* < 0.001), with an *R*^2^ of 0.33. Fourteen of the 24 variables emerged as predictors of future intentions to engage in health precautionary behaviors. The following variables were positively associated with future intentions to engage in health precautionary behaviors: facemask frequency, personal health importance, perceived efficacy of behavioral health precautions, fear of COVID-19 vaccine side-effects, perceived safety of vaccines, general vaccine support, COVID-19 vaccine support, risk perceptions of COVID-19, and perceived severity of COVID-19. Variables that were inversely associated with future intentions to engage in health precautionary behaviors include gender (coded as 1 = Male, 0 = Female), having ever tested positive for COVID-19 (coded as Yes = 1, No = 0), perceived harm of the COVID-19 vaccine, belief that COVID-19 vaccines cause mild-side effects, and vaccine autism myth (see Table 4).

**Table 4:** Summary of multiple linear regression analysis for variables predicting future intentions to engage in health precautionary behaviors (n = 3754).

Variables	*B*	*SE B*	β	*t*	95% confidence intervals		*P*
					Lower	Upper	
Constant	.248	.173		1.430	−.092	.588	.153
Age	.000	.000	.015	1.062	.000	.001	.288
Primary Language (1 = English, 0 = Spanish)	.015	.017	.013	.900	−.018	.048	.368
Gender (1 = Male, 0 = Female)	−.053	.017	−.045	−3.172	−.086	−.020	.002
Ever Tested Positive for COVID-19 (1 = Yes, 0 = No)	−.077	.021	−.051	−3.621	−.119	−.035	<.001
Family or Friend Hospitalized for COVID-19 Complications	.029	.021	.021	1.363	−.013	.070	.173
Family or Friend Deceased Due to COVID-19	−.037	.021	−.027	−1.719	−.079	.005	.086
Essential Worker	.025	.017	.021	1.519	−.007	.058	.129
Facemask Frequency	.328	.027	.167	11.968	.274	.382	<.001
COVID-19 Testing Frequency	.014	.010	.019	1.386	−.006	.034	.166
Personal Health Importance	.076	.011	.098	6.785	.054	.098	<.001
Perceived Susceptibility of COVID-19	.023	.012	.028	1.838	−.002	.047	.066
Perceived Efficacy of Behavioral Health Precautions	.293	.016	.285	18.367	.262	.324	<.001
Perceived Effectiveness of the COVID-19 Vaccine	−.012	.013	−.014	−.910	−.038	.014	.363
Fear of COVID-19 Vaccine Side-Effects	.036	.012	.057	2.925	.012	.060	.003
Perceived Harm of the COVID-19 Vaccine	−.038	.016	−.051	−2.431	−.069	−.007	.015
Belief that COVID-19 Vaccines Cause Mild-Side Effects	−.028	.009	−.043	−2.962	−.047	−.009	.003
Perceived Safety of Vaccines	.029	.014	.035	1.993	.000	.057	.046
Perceived Safety of Moderna COVID-19 Vaccine	.029	.017	.040	1.688	−.005	.064	.092
Perceived Safety of Pfizer COVID-19 Vaccine	−.005	.020	−.006	−.249	−.043	.033	.803
General Vaccine Support	.054	.021	.063	2.560	.013	.095	.010
COVID-19 Vaccine Support	.053	.020	.064	2.625	.013	.092	.009
Vaccine Autism Myth	−.035	.011	−.049	−3.093	−.057	−.013	.002
Risk Perceptions of COVID-19 Infection	.067	.012	.093	5.780	.044	.089	<.001
Perceived Severity of SARS-CoV-2 Infection	.046	.008	.084	5.376	−.092	.588	<.001

*Note*. Sample size is less than 4494 due to missing data. Gender was dichotomized in this analysis as 1 = Male, 0 = Female. Primary language was dichotomized as 1 = English, 0 = Spanish. Ever tested positive for COVID-19 was coded as 1 = Yes, 0 = No.

## 4 Discussion

Several important findings emerged from the current study highlighting the impact of COVID-19 on the border community. Nearly a fifth of the participants reported having tested positive for COVID-19 at some point prior to receiving the vaccine and the most frequently reported symptoms included headache, fatigue, loss of smell, loss of taste, and body aches. These findings are consistent with the most commonly reported symptoms of COVID-19 (Çalıca Utku et al., 2020; Cabrera Martimbianco et al., 2021). Anxiety (19.8%) and depression (12.6%) emerged as symptoms that many individuals attributed to SARS-CoV-2 infection, highlighting the importance of considering mental health when implementing treatments for SARS-CoV-2 infection. Moreover, nearly a quarter of the sample reported that they had a deceased family member due to COVID-19, highlighting the disproportionate impact COVID-19 has on Hispanic/Latinx populations. During the time of the study, the COVID-19 vaccine had not been released for children. However, 79.96% of parents in the sample reported that they intended to vaccinate their child/children with the COVID-19 vaccine. Recent studies have found that parental willingness to vaccinate their children against COVID-19 was strongly associated with parental vaccination status (Sehgal et al., 2023).

Healthcare practitioners, the World Health Organization, and government websites (i.e., CDC and FDA) emerged as the top three trusted sources of information in the current study. A dearth of literature has investigated trustworthy sources of COVID-19 information. Frietze et al. (2023) investigated trusted sources of COVID-19 information in a predominately Hispanic sample and found that the five most trusted and credible sources of information were a health care provider (67.7%), a government website (e.g., CDC, FDA, etc.) (41.8%), the World Health Organization (37.8%), a community health clinic (37.8%), and pharmacists (32.1%). Studies investigating HPV and influenza vaccination have also revealed similar trusted sources of vaccine information (CDC, 2021). These findings have implications for the development of public health campaigns that promote COVID-19 vaccinations to include trusted sources of information such as healthcare practitioners and government websites. Additionally, despite social media being predominately reported as not trusted for COVID-19 information, the current study found that for general health information, the top social media sources participants used included: Google, Facebook, and Instagram. This finding is consistent with a study from 2019 that found that a large portion of participants used social media platforms to find information related to personal health conditions and even receive direct medical consultations online, emphasizing the importance of public awareness to use reputable sources for health information (Sumayyia et al., 2019).

Findings from the regression models highlight the importance of continuing the investigation of theoretical models such as the Health Belief Model (HBM) for predicting behavior, including assessing factors such as perceived severity of COVID-19, perceived susceptibility of COVID-19, risk perceptions of COVID-19, perceived safety of vaccines, and perceived efficacy of behavioral health precautions. These findings are consistent with prior research suggesting that perceived risk, perceived health, perceived severity, and COVID-19 concern are predictors of preventative health behaviors (Shook et al., 2020; Kim and Kim, 2020; Sánchez-Arenas et al., 2021). Findings also reveal an inverse relationship between having ever tested positive for COVID-19 and future intentions to engage in precautionary behaviors. That is, individuals who have tested positive were less likely to engage in precautionary behaviors compared to those who have never tested positive for COVID-19. These findings might be attributed to individuals perceiving themselves to have strengthened immunity against reinfection.

Another important finding is that socio-demographic characteristics such as primary language and gender were predictors of health precautionary behaviors. For example, Model 1 revealed that past health precautionary behavior was higher in individuals who reported that English was their primary language in comparison to those who reported that Spanish was their primary language at home. One potential reason for this observed effect is that Spanish dominant speakers residing in the U.S. might have less access to COVID-19 information in their native language to help guide their precautionary behaviors to prevent COVID-19. Prior research among Latino/Hispanic populations (Alicea-Planas et al., 2021) and other populations (Webster et al., 2020; De Bruin and Bennett, 2020) reported that those who believed themselves to be at risk for COVID-19 were more likely to implement protective behaviors. Public health campaigns should focus on including bilingual COVID-19 materials in both English and Spanish to help increase the awareness of the risks of COVID-19 and behaviors that prevent COVID-19 spread.

Gender differences might contribute to future intentions to engage in COVID-19 health precautionary behaviors as well. For example, males reported slightly lower future intentions to engage in health precautionary behaviors in comparison to females. A few studies have found that women are more likely to be uncertain or unwilling to vaccinate against COVID-19 (Paul et al., 2021; Thunström et al., 2021) and other studies have shown that women are more likely than men to continue to take part in precautionary healthy behavior (Hosen et al., 2021; Kaewpan et al., 2022). Future studies in Hispanics should explore factors that might contribute to understanding gender differences in future intentions to engage in health precautionary behaviors including cultural factors that might be unique to Hispanics such as machismo and marianismo.

The finding that future intentions to engage in health precautionary behaviors was positively associated with fear of COVID-19 vaccine side-effects might suggest that individuals who fear COVID-19 vaccine side-effects are more cautious in nature, thus, more likely to engage in health precautionary behaviors. Perceived harm of the COVID-19 vaccine, belief that COVID-19 vaccines cause mild side effects, and vaccine autism myths were inversely associated in future intentions to engage in health precautionary behaviors in the current study. These findings suggest that negative perceptions and beliefs about vaccines including the COVID-19 vaccine may contribute to lower future intentions to engage in health precautionary behaviors. It might be that individuals who already have negative beliefs about vaccines are more skeptical about health recommendations and thus less likely to engage in health precautionary behaviors. Public health campaigns that focus on changing future intentions to engage in health precautionary behaviors might benefit from correcting the myths and misperceptions associated with vaccines.

Several limitations of the current study should be noted. First, our survey was only administered in English. Having the survey available in Spanish might have increased participation and accuracy in responses given that our sample was predominately Hispanic. Second, our survey was administered to individuals who were in a COVID-19 clinic waiting room immediately after receiving the vaccine. Given that this study occurred during the initial roll out of the COVID-19 vaccine, a range of emotions were likely experienced by participants including anxiety, fear, excitement, and happiness. Our team did not include measures to capture the emotional valence of participants which might have yielded novel findings related to the psychological state of participants immediately after being administered a vaccine during a pandemic. Another limitation of the current study is that we did not provide any type of incentive for participating in the study. Having an incentive contributes to higher engagement and motivation to complete the survey. We believe that having our study advertisements ubiquitously placed throughout the vaccination clinic, the short duration of the survey, and the requirement of participants to remain in the observation room while ensuring no adverse reactions is what contributed to successful recruitment in the current study. Lastly, our study only included vaccinated individuals and thus differences in the beliefs and perceptions between vaccinated and unvaccinated individuals could not be examined.

Establishing the UTEP COVID-19 vaccination clinic played a vital role in enhancing vaccination rates within our predominantly Hispanic and underserved community in El Paso. This initiative served as a crucial stride in diminishing obstacles that hindered access to COVID-19 vaccines. Over thirty-thousand doses of the COVID-19 vaccine were administered and survey data from 4494 vaccinated individuals yielded findings that contribute to understanding the behaviors and perceptions of individuals who are vaccinated during a global pandemic. Theoretical frameworks such as the HBM are useful for understanding past and future precautionary behaviors, however, further research is needed to understand why individuals engage in health precautionary behaviors such as investigating trusted sources of information (e.g., having a recommendation from a healthcare provider versus the CDC), vaccine myths (e.g., vaccines cause autism), and socio-demographic factors (e.g., socioeconomic status). Findings from the current study have implications for informing public health interventions aimed at increasing vaccine uptake and assessing vaccine perceptions.

## Funding

This research did not receive any specific grant from funding agencies in the public, commercial, or not-for-profit sectors.

## CRediT authorship contribution statement

**Gabriel A. Frietze:** Writing – review & editing, Writing – original draft, Supervision, Project administration, Methodology, Investigation, Formal analysis, Data curation, Conceptualization. **Margie E. Padilla:** Writing – review & editing, Writing – original draft, Project administration, Methodology. **Amanda M. Loya:** Writing – review & editing, Writing – original draft. **Alyssa A. Martinez:** Writing – review & editing, Writing – original draft. **Amir G. Hernandez:** Writing – review & editing, Writing – original draft. **José O. Rivera:** Writing – review & editing.

## Declaration of competing interest

None.

## Data Availability

I have shared the link to my data/code at the Attach File step. COVID-19 Clinic Data - Exploring Predictors of COVID-19 Precautionary Behaviors (Original data) (Mendeley Data)
