# Supplementary material for: Exploring predictors of COVID-19 precautionary behaviors during the initial rollout of COVID-19 vaccines in a predominately Hispanic sample
Source: Brain Behav Immun Health. 2024 Sep 19;41:100870. doi: 10.1016/j.bbih.2024.100870 (PMC11465123; doi:10.1016/j.bbih.2024.100870)
Supplement: Multimedia component 1 [file mmc1.doc]

What is your age in years?


	


Please provide your zip code


	


What races do you identify with? (Select all that apply) 
1.	White
2.	Black
3.	Asian
4.	Pacific-Islander
5.	Native-American
6.	Other
7.	Prefer not to answer


Please indicate if you are Hispanic or Latinx.
1.	Non-Hispanic
2.	Hispanic/Latinx/Spanish Descent
3.	Prefer not to answer


Spoken languages (Select all that apply)
1.	English
2.	Spanish
3.	American Sign Language (ASL)
4.	Spanish Sign Language (Lengua de Signos Española, LSE)
5.	Other
6.	Prefer not to answer


Which gender identity do you most identify with?
1.	Male 
2.	Female
3.	Transgender Female
4.	Transgender Male
5.	Gender Variant/Non-Conforming
6.	Prefer not to answer
7.	Other 


What is the primary language spoken at home?
1.	English
2.	Spanish
3.	Other


What is your total combined family income for all members in your household?
1.	Less than $5,000
2.	$5,001 - $20,000
3.	$20,001 - $40,000
4.	$40,001 - $60,000
5.	$60,001 - $80,000
6.	$80,001 - $100,000
7.	$100,001 or more
8.	Do not know
9.	Prefer not to answer


What is your average household size?
1.	One
2.	Two
3.	Three
4.	Four
5.	Five
6.	Six
7.	Seven or more


If you obtain health information online or through social media, what sources do you use? (Select all that apply)
1.	Facebook
2.	Google
3.	Groupme
4.	Instagram
5.	LinkedIn
6.	Reddit
7.	Snapchat
8.	TikTok
9.	Twitter
10.	WebMd
11.	Whatsapp
12.	Yahoo
13.	Other


Have you received the COVID-19 vaccine?
1.	Yes
2.	No


For the following section, you will be asked about your experience with COVID-19.


Do you have any of the following underlying health conditions? If so, please select all that apply.
1.	Autoimmune
2.	Asthma
3.	COPD
4.	Diabetes
5.	High Blood Pressure
6.	Heart Condition
7.	Smoker
8.	Prefer not to answer
9.	I do not have any underlying health conditions
10.	Other


Have you tested positive for COVID-19?
1.	Yes
2.	No


What were your symptoms? (Select all that apply)
1.	Anxiety
2.	Body aches   
3.	Depression
4.	Diarrhea
5.	Difficulty breathing
6.	Dry cough
7.	Fatigue
8.	Fever 
9.	Headache
10.	Loss of smell
11.	Loss of taste 
12.	Nasal congestion
13.	Nausea 
14.	Pink eye
15.	Rash
16.	Sore throat
17.	Vomiting
18.	Other


 How severe were your symptoms for COVID-19?
1.	No symptoms
2.	Slightly severe
3.	Somewhat severe
4.	Extremely Severe


 Where you admitted to the hospital for COVID-19 complications?
1.	Yes
2.	No


 Do you have close family members or friends that have been admitted to the hospital for COVID-19 complications?
1.	Yes
2.	No


Do you have any close family members or friends that have passed away due to COVID-19?
1.	Yes
2.	No


If you have a child, do you intend to get your child/children vaccinated against COVID-19?
1.	Yes
2.	No
3.	Not applicable
4.	Prefer not to answer __________


For the following section, you will be asked about the impact of COVID-19 on employment, food, and utilities.


Are you considered an essential worker/personnel?
1.	Yes
2.	No


Do you and/or your family currently take medications for any health conditions? 
1.	Yes
2.	No


Have you and/or your family been able to purchase your medications?
1.	Yes
2.	No
3.	N/A


Have you received meal assistance from your local _________ : (Select all that apply)
1.	Faith-based Organization 
2.	Family
3.	Friends
4.	Food bank
5.	Non faith-based Organization
6.	UTEP Food pantry
7.	N/A


For the following section, you will be asked about the strategies that you use to prevent the spread of COVID-19.


How often do you wear your facemask when in public places?
1.	Never
2.	Once in a while
3.	About half the time
4.	Most of the time
5.	Always


What are your reasons for not wearing a face mask? (Select all that apply)
1.	Discomfort associated with mask-wearing
2.	Forgetfulness
3.	Ill fit of the facemasks provided
4.	Impracticality of wearing facemasks in certain situations
5.	Lack of motivation
6.	Underlying health condition
7.	Other reasons


How often do you test for COVID-19?
1.	Never
2.	Monthly
3.	Once a week
4.	Twice a week
5.	Three times a week


Please rate whether you agree or disagree with the following statements as they relate to health importance.


I am concerned about my health and am taking action to prevent COVID-19.
1.	Strongly disagree
2.	Disagree
3.	Neutral
4.	Agree
5.	Strongly agree


My health is my top priority.
1.	Strongly disagree
2.	Disagree
3.	Neutral
4.	Agree
5.	Strongly agree


Taking care of my health means a lot to me.
1.	Strongly disagree
2.	Disagree
3.	Neutral
4.	Agree
5.	Strongly agree


Please rate whether you agree or disagree with the following statements as they relate to susceptibility.


I am less likely than most people to get COVID-19.
1.	Strongly disagree
2.	Disagree
3.	Neutral
4.	Agree
5.	Strongly agree


I am not at risk for getting infected with COVID-19.
1.	Strongly disagree
2.	Disagree
3.	Neutral
4.	Agree
5.	Strongly agree


My body could fight off COVID-19 infection.
1.	Strongly disagree
2.	Disagree
3.	Neutral
4.	Agree
5.	Strongly agree


People like me don't get COVID-19.
1.	Strongly disagree
2.	Disagree
3.	Neutral
4.	Agree
5.	Strongly agree


There is little chance that I could get or spread COVID-19 from what I do in my everyday life.
1.	Strongly disagree
2.	Disagree
3.	Neutral
4.	Agree
5.	Strongly agree


Please rate whether you agree or disagree with the following statements as they relate to preventive practices.


Avoiding crowds is an effective method for fighting COVID-19.
1.	Strongly disagree
2.	Disagree
3.	Neutral
4.	Agree
5.	Strongly agree


Practicing social distancing is an effective method for avoiding COVID-19.
1.	Strongly disagree
2.	Disagree
3.	Neutral
4.	Agree
5.	Strongly agree


Staying at home is an effective method for avoiding COVID-19.
1.	Strongly disagree
2.	Disagree
3.	Neutral
4.	Agree
5.	Strongly agree


Washing your hands frequently is an effective method for avoiding COVID-19.
1.	Strongly disagree
2.	Disagree
3.	Neutral
4.	Agree
5.	Strongly agree


Wearing a surgical mask is an effective method for avoiding COVID-19.
1.	Strongly disagree
2.	Disagree
3.	Neutral
4.	Agree
5.	Strongly agree


For the following section, you are being asked about your thoughts about the future COVID-19 vaccine .


If you were receiving information about COVID-19 and the COVID-19 vaccine, who would you believe is the most credible and trustworthy person/place to give you information? (Select all that apply)
1.	Healthcare practitioner (e.g., pediatrician, family practice doctor)
2.	Community health clinic 
3.	Pharmacist
4.	Pharmacy (e.g. Walgreens, CVS, etc.) 
5.	School nurse
6.	Family/Friends
7.	Social media
8.	Social Worker
9.	Government website (e.g., CDC, FDA, etc.) 
10.	World Health Organization (WHO) 
11.	Radio
12.	Television 
13.	Internet
14.	Newspaper
15.	Community health worker or promotor(a) de salud
16.	Elected Officials
17.	Public Health Official
18.	University Communications
19.	Other


If you are paying attention, please select "Blue" for the following response.
1.	Red
2.	Green
3.	Blue
4.	Yellow


For this next set of questions, please indicate if you agree or disagree with the following statements. The statements are in anticipation of a COVID-19 vaccine. 


I believe the COVID-19 vaccine will be effective in preventing the infection.
1.	Strongly disagree
2.	Disagree
3.	Neither agree nor disagree
4.	Agree
5.	Strongly agree


I believe if I get the COVID-19 vaccine, I will be less likely to get sick.
1.	Strongly disagree
2.	Disagree
3.	Neither agree nor disagree
4.	Agree
5.	Strongly agree


I worry that the COVID-19 vaccine might negatively affect me.
1.	Strongly disagree
2.	Disagree
3.	Neither agree nor disagree
4.	Agree
5.	Strongly agree


I worry about the short-term side effects of the COVID-19 vaccine.
1.	Strongly disagree
2.	Disagree
3.	Neither agree nor disagree
4.	Agree
5.	Strongly agree


I worry that the COVID-19 vaccine might have unknown long-term side effects.
1.	Strongly disagree
2.	Disagree
3.	Neither agree nor disagree
4.	Agree
5.	Strongly agree


I think the COVID-19 vaccine may cause health problems in the future.
1.	Strongly disagree
2.	Disagree
3.	Neither agree nor disagree
4.	Agree
5.	Strongly agree


I think the COVID-19 vaccine is unsafe.
1.	Strongly disagree
2.	Disagree
3.	Neither agree nor disagree
4.	Agree
5.	Strongly agree


I think the COVID-19 vaccine might cause short-term problems like fever or discomfort.
1.	Strongly disagree
2.	Disagree
3.	Neither agree nor disagree
4.	Agree
5.	Strongly agree


For the next set of questions, you'll be asked your opinions and behaviors about future COVID-19 vaccines. 


I believe vaccines are safe and effective.
1.	Strongly disagree
2.	Disagree
3.	Neither agree nor disagree
4.	Agree
5.	Strongly agree

I believe vaccines are safe and effective.
1.	Strongly disagree
2.	Disagree
3.	Neither agree nor disagree
4.	Agree
5.	Strongly agree

I believe the Moderna COVID-19 vaccine is safe and effective.
1.	Strongly disagree
2.	Disagree
3.	Neither agree nor disagree
4.	Agree
5.	Strongly agree


I believe the Pfizer COVID-19 vaccine is safe and effective.
1.	Strongly disagree
2.	Disagree
3.	Neither agree nor disagree
4.	Agree
5.	Strongly agree


I believe everyone should be immunized against COVID-19.
1.	Strongly disagree
2.	Disagree
3.	Neither agree nor disagree
4.	Agree
5.	Strongly agree


I believe vaccines cause Autism.
1.	Strongly disagree
2.	Disagree
3.	Neither agree nor disagree
4.	Agree
5.	Strongly agree


My main concern about receiving the Coronavirus (COVID-19) vaccine is: 
1.	Side effects/it would make me sick
2.	Fear of needles/pain
3.	Cultural or religious conflict
4.	Access to vaccine
5.	Lack of money
6.	It wouldn't work
7.	Other


For the following section, you will be asked questions about risk perception of COVID-19. 


I may become infected with COVID-19 more easily than others. 
1.	Strongly disagree
2.	Disagree
3.	Neutral
4.	Agree
5.	Strongly agree


I am afraid to be infected with COVID-19. 
1.	Strongly disagree
2.	Disagree
3.	Neutral
4.	Agree
5.	Strongly agree


For this question, indicate how severe you believe the following statement is. 


How severe do you think the COVID-19 infection is for yourself?
1.	Not severe at all
2.	A little severe
3.	Somewhat severe
4.	Very severe
5.	Extremely severe


Please rate whether you agree or disagree with the following statements as they relate to health precautions.


Over the past three months, I have practiced social distancing to avoid COVID-19.
1.	Strongly disagree
2.	Disagree
3.	Neutral
4.	Agree
5.	Strongly agree


Over the past 3 months, I have stayed at home to avoid COVID-19.
1.	Strongly disagree
2.	Disagree
3.	Neutral
4.	Agree
5.	Strongly agree


Over the past 3 months, I have washed my hands frequently to avoid COVID-19.
1.	Strongly disagree
2.	Disagree
3.	Neutral
4.	Agree
5.	Strongly agree


Over the past 3 months, I wore a mask to avoid COVID-19.
1.	Strongly disagree
2.	Disagree
3.	Neutral
4.	Agree
5.	Strongly agree


In the upcoming 3 months, I intend to practice social distancing to avoid COVID-19.
1.	Strongly disagree
2.	Disagree
3.	Neutral
4.	Agree
5.	Strongly agree


In the upcoming 3 months, I intend to stay at home to avoid COVID-19.
1.	Strongly disagree
2.	Disagree
3.	Neutral
4.	Agree
5.	Strongly agree


In the upcoming 3 months, I intend to wash my hands frequently to avoid COVID-19.
1.	Strongly disagree
2.	Disagree
3.	Neutral
4.	Agree
5.	Strongly agree


In the upcoming 3 months, I intend to wear a mask to avoid COVID-19.
1.	Strongly disagree
2.	Disagree
3.	Neutral
4.	Agree
5.	Strongly agree
